# Investigation of obesity, eating behaviors and physical activity levels living in rural and urban areas during the covid-19 pandemic era: a study of Turkish adolescent

**DOI:** 10.1186/s12887-022-03473-1

**Published:** 2022-07-11

**Authors:** Mehmet Gülü, Hakan Yapici, Elena Mainer-Pardos, Ana Ruivo Alves, Hadi Nobari

**Affiliations:** 1 Department of Coaching Education, Faculty of Sport Sciences, Kirikkale University, Kirikkale, 71450 Turkey; 2 Health Sciences Faculty, Universidad San Jorge, Autovia A23 km 299, 50830 Saragossa, Villanueva de Gállego Spain; 3 Department of Sport Sciences, University of Beira Interior, Covilha, Portugal; 4 Research Center in Sports Sciences, Health Sciences and Human Development, CIDESD, 5001-801 Vila Real, Portugal; 5 Department of Motor Performance, Faculty of Physical Education and Mountain Sports, Transilvania University of Braşov, 500068 Braşov, Romania; 6 Department of Exercise Physiology, Faculty of Educational Sciences and Psychology, University of Mohaghegh Ardabili, Ardabil, 56199-11367 Iran; 7 Faculty of Sport Sciences, University of Extremadura, 10003 Cáceres, Spain

**Keywords:** Childhood obesity, Food addiction, Physical inactivity, Public health, Overweight, Weight management

## Abstract

**Background:**

The purpose of this study was to determine the eating behaviors, obesity and physical activity status of children of similar ages living in rural and urban areas and to examine these relationships during the coronavirus disease (COVID-19) pandemic process.

**Method:**

The research was conducted using the scanning model. The research group consists of children living in rural and urban areas in Turkey. The sample of the study consists of a total of 733 adolescent participants, 351 females (47.9%) and 382 males (52.1%). After anthropometric measurements were made, the Physical Activity Questionnaire for older children and the Yale Food Addiction Scale for children 2.0 were used to determine the food addiction and physical activity status of children during the COVID19 pandemic process. Since the groups were homogeneously distributed, independent samples *t*-test and Pearson correlation test were used.

**Result:**

In terms of food addiction and physical activity levels, children living in the urban have higher scores than children living in rural areas. In addition, children living in the urban were taller and have higher body mass values than those in rural areas. In terms of physical activity level and food addiction levels, while girls living in the urban had higher activity levels than those living in rural areas, no statistically significant difference was found between the physical activity levels of boys. When evaluated in terms of general and gender, it was determined that children living in rural areas were overweight and obese at a higher rate. Obese children had higher levels of food addiction and lower physical activity levels than non-obese children.

**Conclusion:**

In order to prevent childhood obesity, the level of food addiction should be reduced as well as increasing the level of physical activity. This study is limited in terms of cross-sectional evaluation. Future research can experimentally reveal how much obesity is reduced by methods such as exercise and diet interventions.

## Background

Since December 2019 coronavirus disease 2019 (COVID-19) has been classified as a worldwide pandemic. The COVID-19 pandemic has negatively affected life globally in many dimensions. Due to rapid transmission and prolonged incubation period make the containment of COVID-19 very difficult. Therefore, most countries around the world needed to take the “lockdown” option to contain the spread of the virus and people should have stayed-at-home. Consequently, people earned less money, restrictions on transports increased, open-air food markets closed and finally, food products were in short supply at the supermarkets [1, 2]. The sensitive situation caused food insecurity because a sudden drop in supply or access to food. This dramatic event had a negative impact on the children's eating behaviors, increasing their addiction to foods rich in sugar, salt, and fat [3]. Eating-related behaviors are often identified as one of the factors contributing to the development of childhood obesity. What children and adolescents know about eating is important because eating behaviors are determined in childhood, and being obese at this age is a predictor of being obese in adulthood [4, 5]. Overweight and obesity simply occur as an energy imbalance between calories consumed and calories expended [6, 7]. Investigating factors of high energy intake, such as appetite and eating behaviors, may help to understand obesity beyond this basic equation [8–10]. Childhood obesity is one of the most serious public health problems of the twenty-first century [11]. Even a new term “covibesity” has been developed to explain the heightening in obesity rates caused by lockdown during the pandemic [12]. The problem is global and continually affects many low- and middle-income countries, particularly in urban settings [13]. The prevalence is increasing at an alarming rate, the prevalence of overweight and obesity among children and adolescents aged 5–19 has risen dramatically from just 4% in 1975 to just over 18% in 2016. Moreover, the rise overweight has occurred similarly among both boys (19%) and girls (18%) [13]. Almost one all age group in four people in Organization for Economic Cooperation and Development countries are currently obese [14]. The proportion in children is even higher, with one in six children reported to be overweight or obese in developed countries [15]. Early childhood obesity is associated with worse health outcomes and higher healthcare expenditures in adulthood [16]. The risk of obesity-related health problems, particularly ectopic fat accumulation, impaired glucose tolerance, dyslipidemia, hypertension, and diabetes, is also increased for many cardiovascular diseases [6]. In addition, obesity in early childhood is negatively associated with the child's health, quality of life and academic success [17]. Therefore, new healthy strategies are necessary to improve this serious situation in child population.

There are many instruments in the prevention of childhood obesity. Physical activity is important for a healthy life [18]. Increasing the level of physical activity can contribute to better weight management [19]. Numerous cross-sectional studies show a negative relationship between physical activity level and overweight status in school-aged children [20]. Physical activity is the most modifiable factor of energy expenditure, accounting for approximately 25% of total energy expenditure, and therefore, it is a powerful multiplier for influencing the energy balance equation [21]. Physical activity tends to decrease in adolescence [22] according to the World Health Organization (WHO), more than 20% of adolescents lead an inactive life. In addition, the COVID-19 pandemic has caused a decrease in physical activity levels in adolescents [23, 24]. Insufficient physical activity is highlighted as one of the main concerns, especially among socio-economically deprived children [25]. Furthermore, heart disease, osteoporosis and as many disease although the ill effects of appear in adulthood, it is increasingly understood that their development begins in childhood and adolescence [26]. Physical activity is also effective in bone health, mental health and prevention of many diseases [18]. Moreover, physical activity level shows that it is an important factor for the higher prevalence of childhood obesity [27]. Considering all these, it is of great importance to make physical activity a daily routine in childhood.

Over the last decade, interest has risen in the investigation of the importance of the living environment on physical activity and eating behaviors, and consequently the childhood and adolescence obesity [28]. Sallis et al. observed that adolescents who live in urban areas have different lifestyle comparing to adolescents who live a few kilometers from urban centers [29]. Urban children population have a higher prevalence of obesity than rural children [30]. Moreover, urban communities showed higher anthropometric/ body composition indices and lower fitness status than rural communities in children and adolescents [28, 31]. Some studies proposed that nutritional intake and physical activity are influenced by specific living area (e.g., rural vs urban) [32, 33]. As a result, there are a number of reports confirmed that urban areas have higher levels of obesity and lower levels of physical activity than their rural counterparts and this situation has been aggravated by the COVID-19 pandemic.

Childhood overweight is common in upper middle-income countries, while lower-income countries have a lower prevalence [34]. However, while the prevalence rate is increasing faster in low middle-income countries compared to others, overweight seems to increase in almost all countries [35]. There are modifiable differences in obesity rates between rural and urban children. Adolescent overweight and obesity are increasing rapidly in Indonesia, especially with male and gender-specific risks [36]. A study in the United States found significantly higher rates of obesity among rural children [37]. Another study in Ludhiana found that the prevalence of persistent hypertension is increasing in urban areas, even in younger age groups [38]. In addition, obese children often have higher blood pressure compared to thin subjects [38]. In a study conducted in Vietnam, the prevalence of overweight and obesity was found to be higher in urban areas [39]. Urban risk factors identified in the study were being male, consuming large amounts of food, and being indoors for less than 2 h a day. Rural risk factors were found to be frequent consumption of fatty foods [39]. Adolescents' sedentary lifestyles, changing dietary habits, increased fat content of their diets, and decreased physical activity and other factors may affect their obesity and related health problems. Due to the pandemic, it is important to determine the changes in our habits and the reflections of these changes in many areas of our lives like social, nutrition or sporting life. Thus children with limited nutritional knowledge and unhealthy eating habits were five times more likely to be obese [40]. Furthermore, the possible effects of these changes on our eating behaviors, obesity prevalence and physical activity level are not yet known clearly. Therefore, our hypothesis is that an increase in the level of food addiction and a physically inactive life will cause an increase in the prevalence of obesity. We also hypothesized that overweight children would have higher food addiction than underweight and normal-weight children. On the other hand, there are not many studies examining eating behaviors and their relationship with body mass and physical activity in Turkish children with a comprehensive approach. Thus, the aims of this study were 1) to determine the food addiction, obesity and physical activity status of children of similar ages living in rural and urban areas and 2) to examine these relationships during the COVID-19 pandemic process.

## Methods

### Research design and research group

This was an observational retrospective study. The research group consists of 733 adolescent individuals (351 females 47.9%, or 382 males 52.1%) in Turkey. The eligibility criteria consisted of people aged 9–14 years, fluent in Turkish, currently residing in Kirikkale in Turkey and without any mental or chronic illness that prevents them from participating in physical activity. Participants studying in secondary education, and they were invited to work through posters and oral announcements. After reading the information form about the research by the families and children, the participants filled out the questionnaires after measuring their height and weight. Parents were provided with information about the study, objectives, voluntary nature, anonymity, and confidentiality. Finally, informed consents were obtained from the participants and their parents before beginning the investigation. Participants who want to withdraw from the study can leave the study at any time without completing the questionnaire. This study was approved by the Kirikkale University Social and Human Sciences Ethics Committee (12/21.12.2021) in line with the Declaration of Helsinki (2013).

### Data collection method

Descriptive survey model and quantitative method were used in the study [41]. As a result of the reliability analysis of the study, the Cronbach alpha internal consistency reliability coefficient value of the food addiction scale for children was found to be 0.901 (95% confident interval (CI = 0.883–0.917) and the physical activity inventories for children were reliable in the range of 0.77 and was 0.91. The data collection methods consist of three parts: The first part is the personal information form consisting of questions about gender, age and health status. The second part is the evaluation in which anthropometric measurements are made. In the third part, the Physical Activity Questionnaire for Children (PAQ-C) and Yale food addiction scales for children were applied. In order to determine the physical activity status of the participants, the PAQ-C was conducted using the validate Turkish version [42]. In addition, the food addiction scale for children was used to determine the levels of food addiction [43].

### Anthropometric measurements

Height was measured to the nearest 0.1 cm using an anthropometer (Seca 217,

Seca, Hamburg, Germany). During the stature measurement, the child was upright without shoes, with heels together, and with the head on the horizontal plane of Frankfurt. Body mass was measured to the nearest 0.1 kg using a Seca weighing scale (Seca Deutschland Medical Measuring Systems and Scale, Hamburg, Germany) for children dressed in light clothing. Body mass index (BMI) values were calculated using the calculation engine from the Centers for Disease Control and Prevention (CDC) website [44]. Evaluations 85th and 95th percentiles were considered overweight, and those above the 95th percentile were considered obese [45].

### Yale Food Addiction Scale for Children 2.0 (YFAS-C)

Yale Children's Eating Addiction Scale 2.0. it evaluates the eating situation, which has occurred in the form of attacks in children during the last 12 months, and thus food addiction. At the top of this scale, foods with potential for addiction are roughly divided into four groups as follows:

- Desserts such as ice cream, chocolate, cake, cookies, pastry, candy
- Salty snacks like chips, pies and crackers
- Fatty foods such as steak, bacon, burgers, cheeseburgers, pizza and french fries
- Sugary drinks such as soda, lemonade, sports drinks and energy drinks

There are 16 questions prepared according to the DSM-5 diagnostic criteria, which question the thoughts of children about these foods and their experiences in the last year, and the answers to these questions are scored in 5 different ways, from "never" to "always". The severity of food addiction measured by these questions increases with each question. Developed by Ashley Gearhardt ve ark., 2016 [46] and adapted into Turkish by [43].

### Physical Activity Questionnaire for Older Children (PAQ-C)

N Nine out of ten items that make up the physical activity scale are used to calculate activity scores. Item 10 assesses whether the child can continue with normal activities despite being sick or having other interventions in the previous week. However, this item is not included in the calculation of the activity score. The first question in PAQ-C is in the form of an activity checklist describing 22 common leisure and sport activities and another category of 'Other'. Responses to this question are evaluated on the basis of a 5-point rating (1 = no activity, 5 = 7 times or more), from which the average score is calculated; higher scores indicate more physical activity [47]. The clear and unambiguous description of the 22 activities in this questionnaire provides the benefit of a reminder to the respondents. The remaining 8 questions relate to the evaluation of activities performed during the day or at specific time intervals throughout the week (e.g. physical education lesson, recess, lunch, after-school, evening, weekend). These items are scored on a 5-point scale, with higher scores indicating higher activity level. The overall PAQ-C score is obtained by adding the scores for items 1–9, and the final PQ-C activity summary score is the average of the scores for these 9 items. An average of 1 point indicates a low physical activity level and an average of 5 points indicates a high physical activity level [42, 48].

### Data analysis

All data were recorded as mean and standard deviation. Normality was analyzed with the Kolmogorov–Smirnov test and all variables had a normal distribution. Since the kurtosis skewness values are in the range of -2 to + 2, the data are homogeneously distributed. Therefore, binary groups were made. Independent *t*-test was carried out to compare differences between the groups. Pearson correlation coefficients examined associations between BMI and socio-economic status, physical activity levels and food addiction of children in rural and urban area. The following thresholds were used to determine the effect size of the relationships: < 0.1 = trivial; 0.1–0.3 = small; > 0.3–0.5 = moderate; > 0.5–0.7 = large; > 0.7–0.9 = very large; and > 0.9 = nearly perfect [49] Statistical data were analyzed by IBM SPSS version 25 for Windows. Statistical significant was inferred from *p* < 0.05.

## Results

Statistical information about the anthropometric characteristics, physical activity levels and food addiction levels of 733 students studying in urban and rural schools are shown in the Tables 1, 2, 3, 4 and 5. Table 2 determined that the values of the students living in the city among the groups are significantly higher than the values of the students living in the rural areas (*p* < 0.05).

**Table 1:** Descriptive data of children in urban and rural areas

Demographics characteristic	n	%
**Total number of participants**	733	100
**Age**		
11 years	380	51.8
12 years	353	48.2
**Sex**		
Boys	382	52.1
Girls	351	47.9
**Residence**		
Urban	354	48.3
Rural	379	51.7
**BMI (CDC)**		
Overweight	133	18.2
At Risk for overweight	165	22.5
Normal weight	346	47.2
Underweight	89	12.1
**Socioeconomic status groupings**		
High	160	21.8
Medium	321	43.8
Low	252	34.4

*CDC* Disease control and prevention, *BMI* Body mass index

**Table 2:** Statistical information on anthropometric characteristics, physical activity levels and food addiction levels of children living in urban and rural areas

Variables	Urban (*n* = 354)	Rural (*n* = 379)	Total (*n* = 733)	*p*	*Cohen’s d*
**Anthropometric**					
Age (yr)	11.7 ± 0.5	11.3 ± 0.4	11.45 ± 0.5	≤ 0.001*	0.88
Height (cm)	153.9 ± 9.5	150 ± 9.9	152.3 ± 9.8	≤ 0.001*	0.40
Body mass (kg)	49.6 ± 12.5	45.7 ± 12.8	47.6 ± 12.8	≤ 0.001*	0.30
BMI (kg/m2)	20.7 ± 4.1	19.9 ± 4.2	20.3 ± 4.2	≤ 0.006*	0.19
**Physical Activity**					
Total physical activity	2.9 ± 0.8	2.7 ± 0.8	2.8 ± 0.8	≤ 0.002*	0.25
Out of school	2.8 ± 0.7	2.6 ± 0.7	2.8 ± 0.7	≤ 0.002*	0.28
School based	3.6 ± 0.8	3.3 ± 0.9	3.4 ± 0.9	≤ 0.001*	0.35
Food Addiction Score	24.0 ± 9.8	16.8 ± 11.1	20.5 ± 11.1	≤ 0.001*	0.68
**BMI (CDC)**					
Normal Weight	181	165	346	≤ 0.001*	
Underweight	26	63	89	≤ 0.001*	
At risk for overweight	67	98	165	≤ 0.001*	
Overweight	53	80	133	≤ 0.001*	

*BMI* Body mass index, *CDC* Disease control and prevention

^*^p < 0.005

**Table 3:** Statistical information on anthropometric characteristics, physical activity levels and food addiction levels of girls and boys

Variables	Female (*n* = 351)	Male (*n* = 382)	Total (*n* = 733)	*p*	*Cohen’s d*
**Anthropometric**					
Age (yr)	11.8 ± 0.9	11.9 ± 0.8	11.81 ± 0.5	0.256	0.11
Height (cm)	151.9 ± 9.8	152.0 ± 9.8	152.3 ± 9.8	0.280	0.01
Body mass (kg)	47.1 ± 12.7	48.1 ± 12.9	47.6 ± 12.8	0.282	0.07
BMI (kg/m^2^)	20.3 ± 4.1	20.4 ± 4.3	20.3 ± 4.2	0.680	0.02
**Physical Activity**					
Total physical activity	2.8 ± 0.8	2.7 ± 0.8	2.8 ± 0.8	0.999	0.12
Out of school	2.8 ± 0.7	2.8 ± 0.8	2.8 ± 0.7	0.987	0.13
School based	3.3 ± 0.9	3.6 ± 0.9	3.4 ± 0.9	≤ 0.001*	0.33
Food Addiction Score	20.9 ± 10.9	20.17 ± 11.20	20.5 ± 11.1	0.317	0.06
**BMI (CDC)**					
Normal Weight	181	165	346	≤ 0.001*	
Underweight	44	45	89	≤ 0.001*	
At risk for overweight	80	85	165	≤ 0.001*	
Overweight	46	87	133	≤ 0.001*	

*CDC* Disease control and prevention

^*^p < 0.005

**Table 4:** Statistical information on anthropometric characteristics, physical activity levels and food addiction levels of children living in urban and rural areas

*n* = 733	Urban female (*n* = 166)	Urban female (*n* = 185)	*p*	*Cohen’s d*	Rural male (*n* = 188)	Rural male (*n* = 194)	*p Cohen’s d*	
**Anthropometric**								
Age (yr)	11.7 ± 0.46	11.2 ± 0.43	≤ 0.001*	1.12	11.7 ± 0.5	11.3 ± 0.5	≤ 0.001*	0.8
Height (cm)	153.3 ± 9,1	150 ± 10.3	≤ 0.010*	0.33	154.3 ± 9.8	151.2 ± 9.6	≤ 0.003*	0.30
Body mass (kg)	48.4 ± 12,5	45.8 ± 12.7	0.047	0.20	50.6 ± 12.9	45.6 ± 12.9	≤ 0.001*	0.38
BMI (kg/m2)	20.3 ± 3.7	20.1 ± 4.4	0.660	0.04	21.1 ± 4.4	19.6 ± 3.9	≤ 0.001*	0.36
**Physical Activity Level**								
Total physical activity	2.9 ± 0.8	2,6 ± 0.8	≤ 0.007*	0.37	2.8 ± 0.7	2.6 ± 0.8	0.085	0.26
Out of school	2.9 ± 0.8	2,6 ± 0.7	≤ 0.007*	0.39	2.8 ± 0.7	2.6 ± 0.8	0.087	0.26
School based	3.5 ± 0.8	3,1 ± 0.8	≤ 0.001*	0.5	3.6 ± 0.9	3.5 ± 0.9	0.331	0.11
**Food addiction status**								
Food addiction score	17.5 ± 10.9	24.1 ± 9.9	≤ 0.001*	14.4	16.2 ± 11.2	23.9 ± 9.74	≤ 0.001*	
**BMI (CDC)**								
Normal weight	104	77	≤ 0.001*		77	88	≤ 0.001*	
Underweight	13	31	≤ 0.001*		13	32	≤ 0.001*	
At risk for overweight	22	58	≤ 0.001*		40	45	≤ 0.001*	
Overweight	19	27	≤ 0.001*		34	53	≤ 0.001*	
**Sex**	**Female (351)**				**Male (382)**			
Total physical activity	2.7 ± 0.8				2.7 ± 0.8		0.999	
Food addiction score	20.9 ± 10.9				20.1 ± 11.2		0.317	

*CDC* Disease control and prevention, *BMI* Body mass index

^*^p < 0.005

**Table 5:** The relationship between BMI and socio-economic status, physical activity levels and food addiction of students studying in rural and urban children

Variable		r	r^2^	*p* value
**Rural and Urban adolescent**	BMI- socio-economic status	-0.080^a^	0.006	≤ 0.01*
	BMI-food addiction	0.304^a^	0.09	≤ 0.01*
	BMI-Physical activity level	-0.170^a^	0.03	≤ 0.01*
**Urban adolescent**	BMI- socio-economic status	-0.006	0.0004	≤ 0.01*
	BMI-food addiction	0.646^a^	0.42	≤ 0.01*
	BMI-Physical activity level	-0.147^a^	0.02	≤ 0.01*
**Rural adolescent**	BMI- socio-economic status	-0.007	0.0005	> 0.01
	BMI-food addiction	0.084	0.70	> 0.01
	BMI-Physical activity level	-0.226^a^	0.05	≤ 0.01*

^a^Correlation is significant at the 0.01 level (2-tailed)

^*^Correlation is significant at the 0.05 level (2-tailed); BMI: body mass index

When Table 2 was examined, the total physical activity participation of the participants was found to be higher in the urban area than in the rural area (*p* < 0.05*,* effect size = 0.25). It was found that people living in the urban had higher levels of food addiction than those living in the rural area (*p* < 0.01*,* effect size = 0.68). In BMI values, being overweight was found to be higher in those living in rural areas (*p* < 0.01*).*

Table 3 showed that there was a significant statistical difference between in-school physical activity and BMI (*p* < 0.05*,* effect size = 0.33). However, among the anthropometric features of male and female students no statistically significant difference was found (*p* > 0.05) in height (*p* = 0.280), body mass (*p* = 0.282), BMI (*p* = 0.680), total physical activity (*p* = 0.999), total physical activity out of school (*p* = 0.987) and food addiction scores (*p* = 0.317).

There was a significant statistical difference between urban and rural groups in female (Table 4). A statistically significant difference was found between urban and rural groups in terms of anthropometric characteristics of male (*p* < 0.05, effect size = 0.8). However, although it was determined that the physical activity data of students studying in urban and rural schools were numerically better. No statistically significant difference (*p* > 0.05) was found between the groups in terms of total physical activity (*p* = 0.085), total physical activity out of school (*p* = 0.087), total school based physical activity (*p* = 0.331) and Food Addiction Score (*p* = 0.085).

In Table 5, Pearson correlation showed that the highest correlation was observed in food addiction, and the lowest correlation was observed in physical activity level. When we evaluated the children in urban and rural separately, there was no significant relationship between BMI and their socio-economic status (*p* > 0.05). However, significant correlations, low, were observed between BMI and physical activity levels (*p* > 0.05). The highest correlation was observed in food addiction, and the lowest correlation was observed in physical activity values. In addition, it has been determined that there is a negative relationship between food addiction and total physical activity.

When Table 6 was examined, food addiction and physical activity levels of the obese- overweight and underweight-normal weight children were compared. A statistically significant difference was found between the groups (*p* < 0.05).

**Table 6:** Comparison of food addiction and physical activity levels of obese-overweight and underweight-normal weight children

	**BMI**	**n**	**Mean**	**Std. Deviation**	**t**	**Sig. (2-tailed)**
Food addiction	Underweight- Normal weight	435	18.08	9.54	-7.8	≤ 0.001*
	Obese-overweight	298	24.18	12.11		
Physical activity	Underweight- Normal weight	435	2.84	0.82	3.84	≤ 0.001*
	Obese-overweight	298	2.62	0.70		

*BMI* Body mass index

## Discussion

During the COVID-19 pandemic process, many important findings were found in this study, which determined the food addiction, obesity and physical activity status of children of similar ages living in rural and urban areas and examining these relationships in Turkish adolescent. According to the findings of this study, children living in the urban were taller and they have higher body mass values ​​than those living in rural areas. In terms of food addiction and physical activity levels, children living in the urban have higher scores than children living in rural areas. Moreover, female living in the urban had a higher activity level than those living in rural areas, however no statistically significant difference was found between the physical activity levels of male.

In this study, children living in the urban have taller and higher body mass values than those living in rural areas. Similar results were found by Ozdirenç et al. where urban children were taller and heavier than rural children [31]. On the contrary, other study found differences in body composition, with rural children being less weight than urban children [28]. However, in a different study, no significant difference was found between girls and boys in the height (*p* = 0.01) and weight (*p* = 0.07) variables between those living in urban and rural areas [50]. Furthermore, adolescent girls were more likely to be overweight and obese than boys [51, 52]. This may be due to the differences in the physical and social environment in Turkey.

In our study, it was determined that children living in the urban had a higher BMI rate than those living in rural areas. Supporting these findings, Hodgkin et al. found that rural children had significantly lower BMI in New Zealand [28]. In addition, a Chinese and Brazilian study reported a higher prevalence of overweight in children from urban areas [30]. In a similar study, prevalence rates of overweight and obesity were also higher in urban populations compared to rural populations living in Bangladesh [52]. In a study of dietary habits, the average contribution of 'fast food' to total energy intake was significantly higher in the urban area [50]. Prevalence of obesity among Croatian children was high and relevance to the urban/rural environment has not been established [50]. The reason could partly be explained by the unhealthy diet at home during the COVID-19 pandemic due to high intake of fast food", sugary drinks and snacks. Contrary to our findings, in one study, Norwegian children living in rural areas had a higher mean BMI and waist circumference than Norwegian children living in urban areas [53]. Nevertheless, many studies have found a relationship between living in rural areas and being overweight and obese [54–60]. The unfavorable influence of the COVID-19 pandemic and previous results could reflect differences in the economic situation and access to food in these countries. Finally, improvements in diets in urban areas may be the result of healthier diet promote during COVID-19 lockdown, easy access to food and low physical activity levels of children during the study period.

According to the WHO, < 20% of the world's adolescent population lead sufficiently physically active lives [61]. With the COVID-19 pandemic, this situation has worsened, with significant decreases in physical activity levels especially among children living in urban areas [24]. Contrary to these findings, no difference was found between male children living in rural and urban areas in terms of physical activity levels in our study. In addition, in this context, our findings in terms of gender variable, no difference was found between girls and boys. Unlike our findings, some studies in European countries and Kuwait in the literature have found that girls have less physical activity levels than boys [62, 63]. Moreover, girls spent more time sitting and engaging in sedentary activities than boys, which puts them at greater risk for health concerns like metabolic dysregulation or obesity [64, 65]. The fact that these findings present different results from those in our study may be due to regional differences. In some countries, the participation of girls in sports may not be highly encouraged. Therefore, urgent actions are needed to increase physical activity among this population.

The results of this study determined that children with a high level of food addiction tend to be more obese. In relation with this result, Balanzá-Martínez et al. observed that boredom may induce overeating of non-healthier food and increase screen time [66]. Thus, after the "lockdown", daily lifestyle behaviors may have changed, decreasing outdoor time, and increasing sedentary lifestyles among adolescents. In a study conducted in parallel with our findings, overweight children exhibited more food approach behaviors and lower food avoidance behaviors compared to underweight or normal weight children [67]. Sila et al. found a statistically significant difference (*p* > 0.01) in nutritional status between gender, with the percentage of boys being overweight or obese compared to girls [50]. In addition, majority of Kuwaiti adolescents, especially girls, have unhealthy dietary practices [63]. However, no significant difference (*p* > 0.05) was found between girls and boys in our findings. Moreover, Martínez-López et al. revealed an increased in foods rich in fats and sugars among adolescents in Spain, increasing the prevalence of fast-food consumption [68]. These differences between studies may be due to place of residence, education level or the economic situation of the society, access to healthy food and the difference in the kind of food they take.

We are aware of several limitations associated with the small sample size in this study. In addition, if information about the nutritional records of the children included in the study could be accessed, it would be possible to conduct a more detailed analysis. In addition, data were obtained using a scale in addition to body composition measurements in this study. Finally, if the obesity status of the children's families could be examined, it would be possible to predict a hereditary condition.

## Conclusion

This study has produced relevant information about obesity, eating behaviors and physical activity status of Turkish children. The data on obesity and eating behaviors indicate that urban children had a higher height, weight, BMI and food addiction than rural children. However, in terms of physical activity levels no difference was found between children living in rural and urban areas. In addition, children with a high level of food addiction tend to be more obese. The findings highlight the urgent need to implement an appropriate intervention to increase physical activity levels, to improve healthy diet and to decrease sedentary behavior among these populations. In addition, governments should take into consideration participation of girls in sports and implement new effective policies to promote and create adherence healthy behaviors. Finally, to improve this Turkey situation after COVID-19 in children, it would be necessary more regular physical activities and healthy food patterns.

## Data Availability

The datasets used and/or analysed during the current study are available from the corresponding author on reasonable request.

## Abbreviations

COVID-19: Coronavirus disease
BMI: Body mass index
WHO: World Health Organization
PAQ-C: Physical Activity Questionnaire for Older Children
YFAS-C: Yale Food Addiction Scale for Children 2.0
CDC: Disease control and prevention

## References

[1] Das S, Rasul MG, Hossain MS, Khan A-R, Alam MA, Ahmed T, Clemens JD (2020). Acute food insecurity and short-term coping strategies of urban and rural households of Bangladesh during the lockdown period of COVID-19 pandemic of 2020: report of a cross-sectional survey. BMJ Open.

[2] Nobari H, Fashi M, Eskandari A, Villafaina S, Murillo-Garcia Á, Pérez-Gómez J (2021). Effect of COVID-19 on health-related quality of life in adolescents and children: a systematic review. Int J Environ Res Public Health.

[3] Radwan A, Radwan E, Radwan W (2021). Eating habits among primary and secondary school students in the Gaza Strip, Palestine: a cross-sectional study during the COVID-19 pandemic. Appetite.

[4] Brisbois TD, Farmer AP, McCargar LJ (2012). Early markers of adult obesity: a review. Obes Rev.

[5] Blass E (2010). Obesity: causes, mechanisms, prevention, and treatment. The Psychological Record.

[6] Nóbrega C, Rodriguez-López R, editors. Molecular mechanisms underpinning the development of obesity. Switzerland: Springer International Publishing; 2014.

[7] Nobari H, Gandomani EE, Reisi J, Vahabidelshad R, Suzuki K, Volpe SL, Pérez-Gómez J (2022). Effects of 8 Weeks of High-Intensity Interval Training and Spirulina Supplementation on Immunoglobin Levels, Cardio-Respiratory Fitness, and Body Composition of Overweight and Obese Women. Biology.

[8] Webber L, Hill C, Saxton J, Van Jaarsveld CH, Wardle J (2009). Eating behaviour and weight in children. Int J Obes (Lond).

[9] Boswell N, Byrne R, Davies PSW (2018). Eating behavior traits associated with demographic variables and implications for obesity outcomes in early childhood. Appetite.

[10] Indreica E-S, Badicu G, Nobari H (2022). Exploring the correlation between time management, the Mediterranean diet, and physical activity: a comparative study between Spanish and Romanian university students. Int J Environ Res Public Health.

[11] Noncommunicable diseases: Childhood overweight and obesity [https://www.who.int/news-room/questions-and-answers/item/noncommunicable-diseases-childhood-overweight-and-obesity]

[12] Khan MA, Moverley Smith JE (2020). "Covibesity," a new pandemic. Obes Med.

[13] Obesity and overweight [https://www.who.int/news-room/fact-sheets/detail/obesity-and-overweight]

[14] OECD: The Heavy Burden of Obesity: The Economics of Prevention (2019). OECD Health Policy Studies.

[15] Organisation for Economic Co-operation and Development, Obesity Update [http://www.oecd.org/health/obesityupdate.]

[16] Lobstein T, Baur L, Uauy R (2004). Obesity in children and young people: a crisis in public health. Obes Rev.

[17] World Health Organization. Report of the commission on ending childhood obesity. World Health Organization; 2016.

[18] Hallal PC, Victora CG, Azevedo MR, Wells JC (2006). Adolescent physical activity and health: a systematic review. Sports Med.

[19] Swift DL, Johannsen NM, Lavie CJ, Earnest CP, Church TS (2014). The role of exercise and physical activity in weight loss and maintenance. Prog Cardiovasc Dis.

[20] Jiménez-Pavón D, Kelly J, Reilly JJ (2010). Associations between objectively measured habitual physical activity and adiposity in children and adolescents: Systematic review. Int J Pediatr Obes.

[21] Westerterp KR (2017). Control of energy expenditure in humans. Eur J Clin Nutr.

[22] Dumith SC, Gigante DP, Domingues MR, Kohl HW (2011). Physical activity change during adolescence: a systematic review and a pooled analysis. Int J Epidemiol.

[23] Chambonniere C, Lambert C, Fearnbach N, Tardieu M, Fillon A, Genin P, Larras B, Melsens P, Bois J, Pereira B (2021). Effect of the COVID-19 lockdown on physical activity and sedentary behaviors in French children and adolescents: new results from the ONAPS national survey. Eur J Integr Med.

[24] Zenic N, Taiar R, Gilic B, Blazevic M, Maric D, Pojskic H, Sekulic D (2020). Levels and changes of physical activity in adolescents during the COVID-19 pandemic: contextualizing urban vs rural living environment. Appl Sci.

[25] López-Bueno R, López-Sánchez GF, Casajús JA, Calatayud J, Tully MA, Smith L (2021). Potential health-related behaviors for pre-school and school-aged children during COVID-19 lockdown: a narrative review. Prev Med.

[26] Parsons TJ, Power C, Logan S, Summerbell CD (1999). Childhood predictors of adult obesity: a systematic review. Int J Obes Relat Metab Disord.

[27] Ng M, Fleming T, Robinson M, Thomson B, Graetz N, Margono C, Mullany EC, Biryukov S, Abbafati C, Abera SF (2014). Global, regional, and national prevalence of overweight and obesity in children and adults during 1980–2013: a systematic analysis for the Global Burden of Disease Study 2013. The lancet.

[28] Hodgkin E, Hamlin MJ, Ross JJ, Peters F (2010). Obesity, energy intake and physical activity in rural and urban New Zealand children. Rural Remote Health.

[29] Sallis JF, Cerin E, Conway TL, Adams MA, Frank LD, Pratt M, Salvo D, Schipperijn J, Smith G, Cain KL (2016). Physical activity in relation to urban environments in 14 cities worldwide: a cross-sectional study. Lancet.

[30] Wang Y, Monteiro C, Popkin BM (2002). Trends of obesity and underweight in older children and adolescents in the United States, Brazil, China, and Russia. Am J Clin Nutr.

[31] Ozdirenç M, Ozcan A, Akin F, Gelecek N (2005). Physical fitness in rural children compared with urban children in Turkey. Pediatr Int.

[32] Blundell JE, Baker JL, Boyland E, Blaak E, Charzewska J, de Henauw S, Frühbeck G, Gonzalez-Gross M, Hebebrand J, Holm L (2017). Variations in the prevalence of obesity among European countries, and a consideration of possible causes. Obes Facts.

[33] Ujević T, Sporis G, Milanović Z, Pantelić S, Neljak B (2013). Differences between health-related physical fitness profiles of Croatian children in urban and rural areas. Coll Antropol.

[34] WHO (2012). Population-based approaches to childhood obesity prevention.

[35] Alwan A. Global status report on noncommunicable diseases 2010. World Health Organization; 2011.

[36] Agustina R (2021). Meilianawati, Fenny, Atmarita, Suparmi, Susiloretni KA, Lestari W, Pritasari K, Shankar AH: Psychosocial, eating behavior, and lifestyle factors influencing overweight and obesity in adolescents. Food Nutr Bull.

[37] Davis AM, Bennett KJ, Befort C, Nollen N (2011). Obesity and related health behaviors among urban and rural children in the United States: data from the national health and nutrition examination survey 2003–2004 and 2005–2006. J Pediatr Psychol.

[38] Mohan B, Kumar N, Aslam N, Rangbulla A, Kumbkarni S, Sood NK, Wander GS (2004). Prevalence of sustained hypertension and obesity in urban and rural school going children in Ludhiana. Indian Heart J.

[39] Do LM, Tran TK, Eriksson B, Petzold M, Nguyen CTK, Ascher H (2015). Preschool overweight and obesity in urban and rural Vietnam: differences in prevalence and associated factors. Glob Health Action.

[40] Triches RM, Giugliani ERJ (2005). Obesity, eating habits and nutritional knowledge among school children. Rev Saude Publica.

[41] Karasar N. Bilimsel araştırma yöntemi,(31. Basım) Ankara: Nobel Akademik Yayıncılık; 2016.

[42] Erdim L, Ergün A, Kuğuoğlu S (2019). Reliability and validity of the Turkish version of the Physical Activity Questionnaire for Older Children (PAQ-C). Turkish J Med Sci.

[43] Yilmaz E. YFAS 2.0 çocuk versiyonu Türkçe geçerlilik güvenirlik çalışması; 2018.

[44] Centers for Disease Control and Prevention [https://www.cdc.gov/healthyweight/bmi/calculator.html]

[45] Neyzi O, Gunoz H, Furman A, Bundak R, Gokcay G, Darendeliler F, Bas F (2008). Weight, height, head circumference and body mass index references for Turkish children. Cocuk Sagligi ve Hastalikari Dergisi.

[46] Gearhardt AN, Corbin WR, Brownell KD (2016). Development of the Yale food addiction scale version 2.0. Psychol Addict Behav.

[47] Nobari H, Ahmadi M, Pérez-Gómez J, Sá M, Clemente M, Adsuar J, Minasian V, Afonso J (2021). The effect of two types of combined training on bio-motor ability adaptations in sedentary females. The Journal of Sports Medicine and Physical Fitness.

[48] Kowalski KC, Crocker PR, Donen RM (2004). The physical activity questionnaire for older children (PAQ-C) and adolescents (PAQ-A) manual. College of Kinesiology, University of Saskatchewan.

[49] Hopkins WG, Marshall SW, Batterham AM, Hanin J (2009). Progressive statistics for studies in sports medicine and exercise science. Med Sci Sports Exerc.

[50] Sila S, Pavić AM, Hojsak I, Ilić A, Pavić I, Kolaček S (2018). Comparison of obesity prevalence and dietary intake in school-aged children living in rural and urban area of Croatia. Prev Nutr Food Sci.

[51] Otitoola O, Oldewage-Theron W, Egal A (2021). Prevalence of overweight and obesity among selected schoolchildren and adolescents in Cofimvaba, South Africa. South African Journal of Clinical Nutrition.

[52] Banik S, Rahman M (2018). Prevalence of overweight and obesity in Bangladesh: a systematic review of the literature. Curr Obes Rep.

[53] Biehl A, Hovengen R, Grøholt EK, Hjelmesæth J, Strand BH, Meyer HE (2013). Adiposity among children in Norway by urbanity and maternal education: a nationally representative study. BMC Public Health.

[54] Moraeus L, Lissner L, Yngve A, Poortvliet E, Al-Ansari U, Sjöberg A (2012). Multi-level influences on childhood obesity in Sweden: societal factors, parental determinants and child's lifestyle. Int J Obes (Lond).

[55] Lutfiyya MN, Lipsky MS, Wisdom-Behounek J (2007). Inpanbutr-Martinkus M: is rural residency a risk factor for overweight and obesity for U.S. children?. Obesity (Silver Spring).

[56] Liu J, Bennett KJ, Harun N, Probst JC (2008). Urban-rural differences in overweight status and physical inactivity among US children aged 10–17 years. J Rural Health.

[57] Bruner MW, Lawson J, Pickett W, Boyce W, Janssen I (2008). Rural Canadian adolescents are more likely to be obese compared with urban adolescents. Int J Pediatr Obes.

[58] Thorisdóttir IE, Kristjansson AL, Sigfusdottir ID, Allegrante JP (2012). The landscape of overweight and obesity in Icelandic adolescents: geographic variation in body-mass index between 2000 and 2009. J Community Health.

[59] Ismailov RM, Leatherdale ST (2010). Rural-urban differences in overweight and obesity among a large sample of adolescents in Ontario. Int J Pediatr Obes.

[60] Sjöberg A, Moraeus L, Yngve A, Poortvliet E, Al-Ansari U, Lissner L (2011). Overweight and obesity in a representative sample of schoolchildren - exploring the urban-rural gradient in Sweden. Obes Rev.

[61] World Health Organization. Prevalence of insufficient physical activity. Global Health Observatory (GHO) data. 2010.

[62] Verloigne M, Loyen A, Van Hecke L, Lakerveld J, Hendriksen I, De Bourdheaudhuij I, Deforche B, Donnelly A, Ekelund U, Brug J, van der Ploeg HP (2016). Variation in population levels of sedentary time in European children and adolescents according to cross-European studies: a systematic literature review within DEDIPAC. Int J Behav Nutr Phys Act.

[63] Allafi A, Al-Haifi AR, Al-Fayez MA, Al-Athari BI, Al-Ajmi FA, Al-Hazzaa HM, Musaiger AO, Ahmed F (2014). Physical activity, sedentary behaviours and dietary habits among Kuwaiti adolescents: gender differences. Public Health Nutr.

[64] Raynor HA, Bond DS, Freedson PS, Sisson SB (2012). Sedentary behaviors, weight, and health and disease risks. Journal of obesity.

[65] Dempsey PC, Owen N, Biddle SJ, Dunstan DW (2014). Managing sedentary behavior to reduce the risk of diabetes and cardiovascular disease. Curr Diab Rep.

[66] Balanzá-Martínez V, Atienza-Carbonell B, Kapczinski F, De Boni RB (2020). Lifestyle behaviours during the COVID-19–time to connect. Acta Psychiatrica Scandinavica.

[67] Spence JC, Carson V, Casey L, Boule N (2011). Examining behavioural susceptibility to obesity among Canadian pre-school children: the role of eating behaviours. Int J Pediatr Obes.

[68] Martín-Rodríguez A, Tornero-Aguilera JF, López-Pérez PJ, Clemente-Suárez VJ (2022). Dietary patterns of adolescent students during the COVID-19 pandemic lockdown. Physiol Behav.

